# Loss of BMP2 and BMP4 Signaling in the Dental Epithelium Causes Defective Enamel Maturation and Aberrant Development of Ameloblasts

**DOI:** 10.3390/ijms23116095

**Published:** 2022-05-29

**Authors:** Claes-Göran Reibring, Maha El Shahawy, Kristina Hallberg, Brian D. Harfe, Anders Linde, Amel Gritli-Linde

**Affiliations:** 1 Department of Oral Biochemistry, Institute of Odontology, Sahlgrenska Academy at the University of Gothenburg, SE-40530 Göteborg, Sweden; claesreibring@yahoo.se (C.-G.R.); maha.elshahawy@minia.edu.eg (M.E.S.); kristina.hallberg@odontologi.gu.se (K.H.); linde@odontologi.gu.se (A.L.); 2 Department of Oral Biology, Faculty of Dentistry, Minia University, Minia 61511, Egypt; 3 Department of Molecular Genetics and Microbiology Genetics Institute, College of Medicine, University of Florida, Gainesville, FL 32610, USA; bharfe@ufl.edu

**Keywords:** amelogenesis imperfecta, alkaline phosphatase, bone morphogenetic proteins, cell-cell adhesion, cell-matrix attachment, CRE/LoxP, ezrin/radixin/moesin, stratum intermedium, pathological cell migration, ruffled plasma membrane

## Abstract

BMP signaling is crucial for differentiation of secretory ameloblasts, the cells that secrete enamel matrix. However, whether BMP signaling is required for differentiation of maturation-stage ameloblasts (MA), which are instrumental for enamel maturation into hard tissue, is hitherto unknown. To address this, we used an in vivo genetic approach which revealed that combined deactivation of the *Bmp2* and *Bmp4* genes in the murine dental epithelium causes development of dysmorphic and dysfunctional MA. These fail to exhibit a ruffled apical plasma membrane and to reabsorb enamel matrix proteins, leading to enamel defects mimicking hypomaturation amelogenesis imperfecta. Furthermore, subsets of mutant MA underwent pathological single or collective cell migration away from the ameloblast layer, forming cysts and/or exuberant tumor-like and gland-like structures. Massive apoptosis in the adjacent stratum intermedium and the abnormal cell-cell contacts and cell-matrix adhesion of MA may contribute to this aberrant behavior. The mutant MA also exhibited severely diminished tissue non-specific alkaline phosphatase activity, revealing that this enzyme’s activity in MA crucially depends on BMP2 and BMP4 inputs. Our findings show that combined BMP2 and BMP4 signaling is crucial for survival of the stratum intermedium and for proper development and function of MA to ensure normal enamel maturation.

## 1. Introduction

In mammals, enamel, the hard tissue that covers the tooth crown, is produced by specialized cells called ameloblasts. Ameloblasts perform various important functions including ion and water transport as well as secretion of proteins and enzymes to ensure proper formation of enamel crystal structures. Ameloblasts derive from progenitors constituting the inner dental epithelium, a layer of proliferating cells adjacent to the dental papilla mesenchyme. The dental papilla mesenchyme gives rise to odontoblasts which produce predentin and dentin matrices as well as to cells that form the dental pulp. The inner dental epithelium and ameloblasts are part of the enamel organ, a tissue of ectodermal origin which also comprises cells of the stratum intermedium, the stellate reticulum and the outer dental epithelium.

Enamel formation (amelogenesis) is a process that occurs in three stages: the secretory stage, the transition stage, and the maturation stage [1,2]. Soon after predentin is produced by odontoblasts, preameloblasts are induced to differentiate into secretory ameloblasts by signals emanating from predentin and odontoblasts [3,4,5,6]. Secretory ameloblasts are highly polarized tall cells, characterized by an apical cellular extension known as Tomes’ process [2,7]. During the secretory stage, the enamel matrix is a soft substance containing immature calcium hydroxyapatite crystallites, enzymes, and enamel matrix proteins (EMPs). Secretory ameloblasts secrete the majority of EMPs. These include amelogenins, the most abundant EMPs, ameloblastin (*alias* amelin in rats [7]) and enamelin [7,8,9].

Secretory ameloblasts also produce matrix metalloproteinase-20 (MMP20; enamelysin) [10,11,12], a secreted zinc-dependent endopeptidase, which cleaves enamel proteins into smaller peptides soon after their secretion [8,12,13]. MMP20 proteolytic processing of EMPs at the secretory stage enables calcium hydroxyapatite crystallites to grow in width and thickness [8].

The relatively brief transition stage of amelogenesis begins when the secreted enamel matrix attains its full thickness and volume. During the transition stage secretory ameloblasts undergo drastic morphological changes characterized by loss of Tomes’ processes and decreased height [1,2,14,15]. Subsequently, transition-stage ameloblasts differentiate into maturation-stage ameloblasts, the function of which is critical for proper enamel maturation [2].

Maturation-stage ameloblasts undergo repetitive cycles during which they alternate between two histologically and functionally distinct cell types, known as ruffle-ended and smooth-ended ameloblasts, until the enamel is mature [2,16]. Ruffle-ended ameloblasts display an infolded apical plasma membrane, whereas smooth-ended ameloblasts lack apical membrane infolding [2,16].

During the secretory and transition stages, the stratum intermedium forms a distinct layer of cuboidal epithelial cells adjacent to ameloblasts [2]. During the maturation stage, cells of the stratum intermedium which remain adjacent to ameloblasts form, together with cells of the stellate reticulum and outer dental epithelium, a convoluted papillary layer penetrated by prominent vascular loops [2,17,18].

During the transition stage of amelogenesis, 25% of the ameloblast population undergoes programmed cell death through apoptosis, and 25% of the remaining ameloblasts perish by apoptosis at later stages of tooth formation [7,19,20].

Transition-stage and early maturation-stage ameloblasts express low levels of amelogenin mRNA and protein [21,22,23,24,25,26], and at the maturation stage proper ameloblasts cease to produce amelogenin mRNA [26]. By contrast, high levels of ameloblastin are produced by transition-stage and all maturation-stage ameloblasts [22,27]. These cells secrete other proteins as well, including amelotin and odontogenic ameloblast-associated protein/apin [28,29,30,31,32].

From the onset of the transition stage of amelogenesis onwards, ameloblasts express and secrete high levels of kallikrein-related peptidase 4 (KLK4) [11,33,34], a serine protease which degrades EMPs [35]. *Mmp20* expression levels decline in ameloblasts during the transition and early maturation stages, and *Mmp20* mRNA is barely detectable in late maturation-stage ameloblasts [11,36].

The importance of EMPs, MMP20, and KLK4 for normal enamel formation has been revealed by genetic studies both in humans [9,36,37] and in mouse models [26,38,39,40,41,42,43,44,45,46,47,48,49,50,51].

Enamel maturation into a highly mineralized tissue [7] requires substantial removal of water and EMPs. Secretory and maturation-stage ameloblasts seem to remove EMP degradation products and likely also nearly intact EMPs from the enamel matrix by endocytosis, and the enamel matrix clearance phenomenon becomes prominent at the maturation stage of amelogenesis with increased resorptive activity of maturation-stage ameloblasts [52,53].

Inherited defects of dental enamel, known as *amelogenesis imperfecta*, occur in isolation or as part of a syndrome. The disorders affect the quality and/or the quantity of enamel in all teeth of the primary and permanent dentitions. Defects of enamel development during the secretory stage leads to hypoplastic amelogenesis imperfecta characterized by either total agenesis of enamel or formation of an abnormally thin enamel which is otherwise normally mineralized. Defects occurring during the maturation stage of amelogenesis cause hypomineralized amelogenesis imperfecta characterized by the development of enamel of normal thickness but of poor quality. This category comprises two phenotypes, one is known as hypomaturation amelogenesis imperfecta which is caused by failure of the removal of EMPs from the developing enamel, and the other is, the so-called, hypocalcified amelogenesis imperfecta which results from defective transport of calcium ions into enamel [9].

Normal enamel formation crucially depends on proper differentiation and function of ameloblasts. Several factors, including the signaling molecules bone morphogenetic proteins (BMPs) and sonic hedgehog (SHH), as well as transcription factors such as MSX2 and RUNX2, are critical for normal tooth development [54,55,56,57,58,59]. In the ameloblast lineage, it has been shown that SHH and BMP signaling pathways are required for differentiation of secretory ameloblasts [54,55,57], and that BMPs, notably BMP2 and BMP4 [58], as well as RUNX2 [59], are crucial for enamel maturation.

BMPs are secreted ligands belonging to the transforming growth factor β family, known to exert multi-faceted crucial functions during embryonic development and postnatal homeostasis [60,61]. BMPs have been classified into different groups, among which the DPP group includes BMP2, BMP4, and *Drosophila* decapentaplegic (DPP), and the 60A group consists of BMP5, BMP6, BMP7, and BMP8 and *Drosophila* 60A/glass bottom boat [62].

During tooth morphogenesis, *Bmps* exhibit dynamic expression patterns [57,63]. At later developmental stages, secretory ameloblasts express moderate levels of *Bmp2* and *Bmp7* and high levels of *Bmp4* and *Bmp5* [57,63], whereas maturation-stage ameloblasts express high levels of *Bmp4* and *Bmp7* and relatively lower levels of *Bmp2* [64]. The dental mesenchyme and its derivatives also express *Bmps*. During early stages of tooth development, the dental papilla and odontoblasts express *Bmp2* and *Bmp4*, and during later stages of odontogenesis odontoblasts, the pulp and dental sac mesenchyme express the *Bmp2*, *Bmp4*, *Bmp5*, and *Bmp7* genes [57,63,64].

In mouse incisors, which form enamel solely on their buccal tooth crown-analog side, total abrogation of BMP signaling in the dental epithelium through overexpression of follistatin, a BMP inhibitor, prevents ameloblast differentiation and enamel formation, whereas loss of follistatin causes ectopic ameloblast differentiation and enamel formation in their lingual root analog side [57]. More recently, it has been shown that Keratin14-CRE (K14-CRE)-mediated deactivation of the *Bmp2* and *Bmp4* genes in the dental epithelium impinges upon enamel maturation, a defect that has been suggested to be secondary to diminished production of MMP20 and KLK4 by maturation-stage ameloblasts [58]. However, whether loss of *Bmp2* and *Bmp4* has a negative impact on maturation-stage ameloblast differentiation and function remained an unanswered question.

To address this question, we generated mice with combined loss-of-function of the *Bmp2* and *Bmp4* genes specifically in the dental epithelium through ShhGFPCRE-mediated irreversible gene deactivation and assessed the molecular and cellular changes caused by epithelial loss of function of *Bmp2* and *Bmp4*. Our study reveals that, in addition to exhibiting impaired enamel maturation, the mutant teeth developed morphologically and functionally abnormal maturation-stage ameloblasts. Furthermore, subsets of mutant maturation-stage ameloblasts aberrantly underwent collective and/or single cell migration away from the enamel surface, forming cysts and nodules resembling tumors. We show that this migration of maturation-stage ameloblast is likely caused by their abnormal cell-cell contacts and cell-matrix attachment and by massive apoptosis in the adjacent stratum intermedium.

## 2. Results

### 2.1. Normal Tooth Development before the Maturation Stage of Amelogenesis upon Combined Loss of Bmp2 and Bmp4 Gene Function in the Dental Epithelium

To determine the role of BMP2 and BMP4 signaling in ameloblast differentiation and function, we generated *ShhGFPCRE/Bmp2^f/f^/Bmp4^f/f^* and *ShhGFPCRE/Bmp2^+/f^/Bmp4^f/f^* double mutant mice in which irreversible deletion of the floxed (f) alleles of *Bmp2* and *Bmp4* occurs specifically in the dental epithelium as *Shh* is expressed exclusively in dental epithelial cells [54,55,65,66,67,68,69]. Indeed, during tooth development, *Shh* is expressed in the dental placode, enamel knots, stratum intermedium, and the stellate reticulum, as well as in the inner dental epithelium and its derivatives, the pre-ameloblasts and secretory ameloblasts [54,55,65,66,67,68,69].

In the *ShhGFPCRE/Bmp2^f/f^/Bmp4^f/f^* and *ShhGFPCRE/Bmp2^+/f^/Bmp4^f/f^* double mutant mice, epithelial cells that express or have expressed *Shh* are expected to undergo irreversible ShhGFPCRE-mediated deactivation of the floxed *Bmp2* and *Bmp4* alleles. Accordingly, β-galactosidase histochemistry which visualizes CRE activity in sections from *ShhGFPCRE/R26R* reporter mice, revealed dynamic distribution patterns of CRE activity in the dental epithelium of developing teeth (Figure S1A–G’).

At advanced developmental stages of the *ShhGFPCRE/R26R* teeth, cells of the dental epithelium, including secretory and maturation-stage ameloblasts, displayed robust CRE activity (Figure S1G,G’), and this was similar to the distribution patterns and intensity of CRE activity in *K14-CRE/R26R* teeth in which dental epithelial cells that express or have expressed Keratin-14 exhibit CRE activity (Figure S1H,H’). Furthermore, in situ hybridization with oligonucleotide probes targeting the deleted alleles of *Bmp2* and *Bmp4* confirmed ShhGFPCRE-mediated *Bmp2* and *Bmp4* gene deletion in ameloblasts (Figure S2). In situ hybridization also revealed that, in control teeth, the stratum intermedium and papillary layer express *Bmp2* and *Bmp4*, albeit at lower levels than ameloblasts, and that in the mutant teeth, these cell layers lost *Bmp2* and *Bmp4* expression (Figure S2). As expected, *Bmp2* and *Bmp4* expression in the dental mesenchyme of the mutant teeth was unaffected (Figure S2).

As a first step towards assessing the impact of loss of BMP2 and BMP4 signaling on ameloblasts, we processed control and mutant teeth for histology. Alcian blue van-Gieson staining of tooth sections revealed normal histological features during tooth morphogenesis and during the secretory stage of amelogenesis in the *ShhGFPCRE/Bmp2^f/f^/Bmp4^f/f^* and *ShhGFPCRE/Bmp2^+/f^/Bmp4^f/f^* mutants (Figure S3A–F’). However, during enamel maturation, the *ShhGFPCRE/Bmp2^f/f^/Bmp4^f/f^* and *ShhGFPCRE/Bmp2^+/f^/Bmp4^f/f^* mutants displayed several anomalies. In sections across postnatal demineralized teeth at the level of the maturation-stage of amelogenesis, mature enamel disappears, leaving a space between dentin and maturation-stage ameloblasts, a phenomenon that was observed in control teeth as expected (Figure S3G,G’). However, sections of mutant teeth showed persistence of enamel matrix, indicating failure of proper enamel maturation (Figure S3H–I’). As predicted by the patterns of ShhGFPCRE activity (Figure S1) and the expression of the deleted *Bmp2* and *Bmp4* alleles (Figure S2), the mutant teeth did not exhibit defects in the dental mesenchyme-derived tissues (Figures S2 and S3; see also Figure S4).

Strikingly, in the mutant teeth, the surface of the enamel matrix was wavy, and multicellular structures/nodules encompassing an enamel-like extracellular substance were found outside of the ameloblast layer (Figure S3I’); these nodules, which were also found in incisors as described below, could harbor ectopically located maturation-stage ameloblasts that have emigrated away from the ameloblast layer, as no disorganization of the ameloblast layer was observed before the maturation stage (Figure S3).

**Figure 1:**
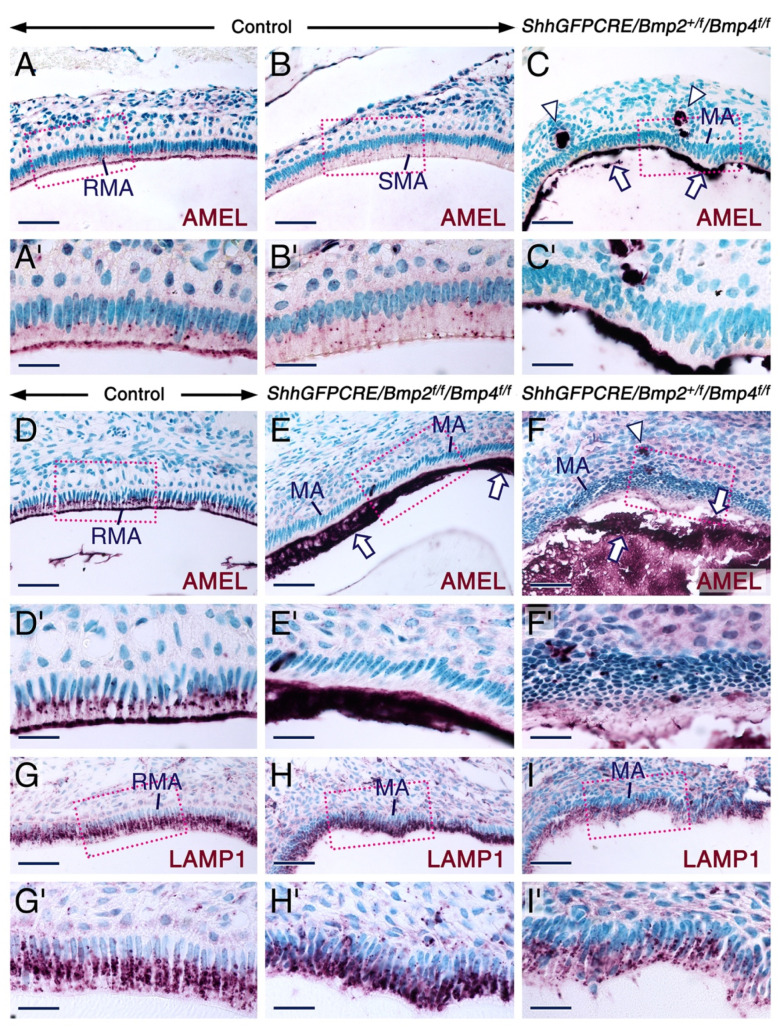
Combined loss of BMP2 and BMP4 signaling in the dental epithelium causes abnormal enamel maturation and aberrant development of maturation-stage ameloblasts. (**A**–**I**) Sections across incisors (**A**–**C**) and molars (**D**–**I**) at the level of maturation-stage of amelogenesis from adult (**A**–**C**) and 12 days postpartum (**D**–**I**) control (**A**,**B**,**D**,**G**), *ShhGFPCRE/Bmp2^+/f^/Bmp4^f/f^* (**C**,**F**,**I**) and *ShhGFPCRE/Bmp2^f/f^/Bmp4^f/f^* (**E**,**H**) mice. (**A**–**F**) Representative amelogenin (AMEL) immunostaining (purple) in parasagittal (**D**–**F**) and frontal (**G**–**H**) sections across molars and oblique (**A**–**C**) sections across incisors showing retained amelogenin-positive enamel matrix in the mutant teeth (arrows in (**C**,**E**,**F**)). In control teeth, (**A**,**B**,**D**) amelogenin immunostaining is concentrated in intracytoplasmic vesicles in both the ruffle-ended (RMA) and smooth-ended (SMA) maturation-stage ameloblasts and in the ruffled apical plasma membrane of RMA. The mutant maturation-stage ameloblasts (**C**,**E**,**F**) fail to exhibit an amelogenin-positive apical border and amelogenin-positive intracytoplasmic vesicles. The mutant maturation-stage ameloblasts are severely misshapen and show altered cell polarity with more apically-located nuclei. The mutant teeth also show nodules encompassing an amelogenin-positive enamel-like substance (arrowheads in (**C**,**F**)). (**A’**–**F’**) are magnified views of the boxed areas in (**A**–**F**). (**G**–**I**) Representative immunostaining (purple) for LAMP1 showing the presence of LAMP1-positive intracellular vesicles in the mutant maturation-stage ameloblasts. (**G’**–**I’**) are magnified views of the boxed areas in (**G**–**I**). Note the abnormal attachment of the apical border of mutant maturation-stage ameloblasts to the enamel matrix (**H**–**I’**). MA, maturation-stage ameloblasts; RMA, ruffle-ended maturation stage ameloblasts; SMA, smooth-ended maturation-stage ameloblasts. Scale bars: 50 µm (**A**–**I**) and 20 µm (**A’**–**I’**).

**Figure 2:**
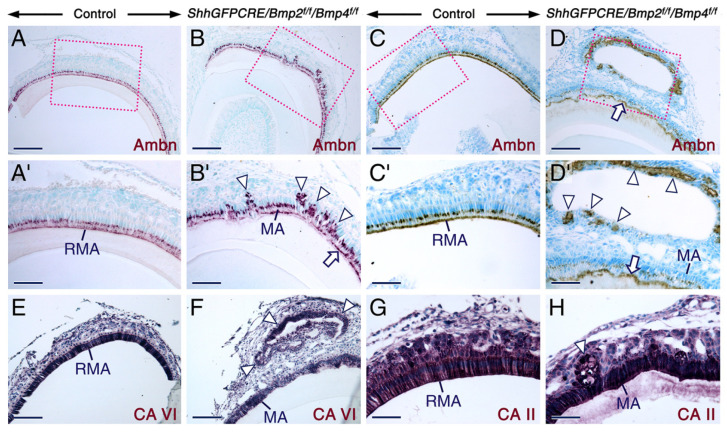
Enamel defects and abnormal development of maturation-stage ameloblasts upon combined loss of BMP2 and BMP4 signaling. (**A**–**H**) Sections across incisors at the level of early maturation-stage (**A**,**B**) and maturation stage (**C**–**H**) of amelogenesis from adult control (**A**,**C**,**E**,**G**) and *ShhGFPCRE/Bmp2^f/f^/Bmp4^f/f^* (**B**,**D**,**F**,**H**) mice. (**A**–**D**) Representative immunostaining (purple or brown) for ameloblastin (Ambn). (**A’**–**D’**) are magnified views of the boxed areas in (**A**–**D**). In the mutant teeth ameloblastin is detectable at the surface of the retained enamel matrix (arrows in (**B’**,**D**) and (**D’**)) and is expressed by maturation-stage ameloblasts, including those that formed nodules and cyst-like structures outside of the ameloblast layer (arrowheads in (**B’**,**D’**)). (**E**–**H**) Representative immunostaining (purple) for carbonic anhydrase VI (CAVI; (**E**,**F**)) and carbonic anhydrase II (CAII; (**G**,**H**)). In the mutant teeth maturation-stage ameloblasts, including those that formed nodules (arrowheads in (**F**,**H**)), express CAVI and CAII. MA, maturation-stage ameloblasts; RMA, ruffle-ended maturation stage ameloblasts. Scale bars: 100 µm (**A**–**D**,**E**,**F**) and 50 µm (**G**,**H**).

Retention of enamel proteins at the maturation-stage and after tooth eruption is a pathognomonic sign of enamel hypomaturation [9,44]. We found that, at later postnatal stages, the erupted molars of the *ShhGFPCRE/Bmp2^f/f^/Bmp4^f/f^* and *ShhGFPCRE/Bmp2^+/f^/Bmp4^f/f^* mutants exhibited severe abrasion of the tooth crown and persistence of amelogenin-containing enamel matrix (Figure S4A–F). Immunostaining of sections across mutant postnatal incisors and molars at the level of maturation-stage ameloblasts revealed the presence of amelogenin (Figure 1A–F’) and ameloblastin (Figure 2A-D’) proteins in the retained enamel matrix. In the mutant teeth, amelogenin was also detectable in the enamel matrix-like substance encompassed by cells that formed nodules outside of the maturation-stage ameloblast layer (Figure 1C,F). That these nodules contain maturation-stage ameloblasts that emigrated from the enamel surface was evidenced by immunostaining for ameloblastin, carbonic anhydrase VI and carbonic anhydrase II (Figure 2A–H), as these proteins are known to be abundantly expressed in maturation-stage ameloblasts [22,27,70,71,72,73,74]. In the mutant teeth, maturation-stage ameloblasts that apparently underwent collective cell migration away from the ameloblast layer were severely shrunken and formed, together with other cells of the dental epithelium, multicellular structures (Figure 1C,F and Figure 2B,D,F,H; see also Figure S5). While some of these structures encompassed an amelogenin-positive extracellular matrix (Figure 1C,F), others were not associated with extracellular material and formed more elaborate tumor-like and gland-like structures as well as cysts (Figure 2B,D,F,H and Figure S5C–F’).

### 2.2. Combined Loss of BMP2 and BMP4 Signaling in the Dental Epithelium Causes Development of Dysfunctional Maturation-Stage Ameloblasts Lacking a Ruffled Border and Exhibiting Altered Cell-Cell and Cell-Matrix Contacts

Normal enamel maturation requires removal of EMPs from the developing enamel, and maturation-stage ameloblasts have been implicated in the reabsorption of degraded EMPs at the maturation stage of amelogenesis [52,53]. In maturation-stage ameloblasts, amelogenin immunoreactivity has been shown to be strong in multivesicular bodies and in the ruffled apical pole, while it is weak in cellular organelles involved in the secretory pathway [23]. In the control teeth, maturation-stage ameloblasts exhibited amelogenin-positive intracytoplasmic vesicles and amelogenin-positive apical ruffled border (Figure 1A–B’,D,D’). As maturation-stage ameloblasts produce minute amounts of amelogenins during the maturation stage proper [23,26], it is highly likely that the amelogenin-positive intracytoplasmic vesicles and ruffled border seen in the control maturation-stage ameloblasts contain reabsorbed amelogenin degradation products. By contrast, the mutant maturation-stage ameloblasts failed to display amelogenin immunoreactive intracytoplasmic vesicles and ruffled borders (Figure 1C,C’,E,E’,F,F’). These data suggest that in the mutant teeth, maturation-stage ameloblasts have defective endocytotic function, leading to abnormal enamel formation.

To determine whether the lack of amelogenin-positive intracellular vesicles in the mutant maturation-stage ameloblasts is caused by abnormal development of vesicles such as endosomes and lysosomes, we carried out immunostaining for lysosome-associated membrane protein 1 (LAMP1), a protein expressed in the endosomal/lysosomal system [75,76,77], and found no alterations in LAMP1-positive vesicles in the mutant maturation-stage ameloblasts (Figure 1G–I’). This finding shows that the absence of amelogenin-positive intracellular vesicles in the mutant maturation-stage ameloblasts is not caused by anomalies in the endosomal/lysosomal system, and strongly suggests that the abnormal development of the apical membranes is the cause of defective endocytotic activity of these cells.

ERM proteins, namely ezrin (also known as cytovillin and villin-2), radixin, and moesin (membrane organizing extension spike protein), are three vertebrate paralogues with key biological functions. These proteins are important for the organization of the cortical skeleton by linking the actin microfilament network to the apical membrane of epithelial cells [78,79]. They are involved in controlling cell survival, epithelial cell integrity [79], and other cellular processes, including endocytosis and vesicular trafficking [80], membrane transport of electrolytes, cell adhesion, and membrane ruffling, as well as the formation of microvilli and filopodia [81]. ERMs are activated through various processes, one of which is phosphorylation of conserved threonine residues at their C-terminal domain [78,79,80]. In maturation-stage ameloblasts, ERM immunoreactivities have been shown to concentrate in the apical, ruffled border of ruffle-ended maturation-stage ameloblasts, and in the lateral membranes of smooth-ended maturation-stage ameloblasts [82].

**Figure 3:**
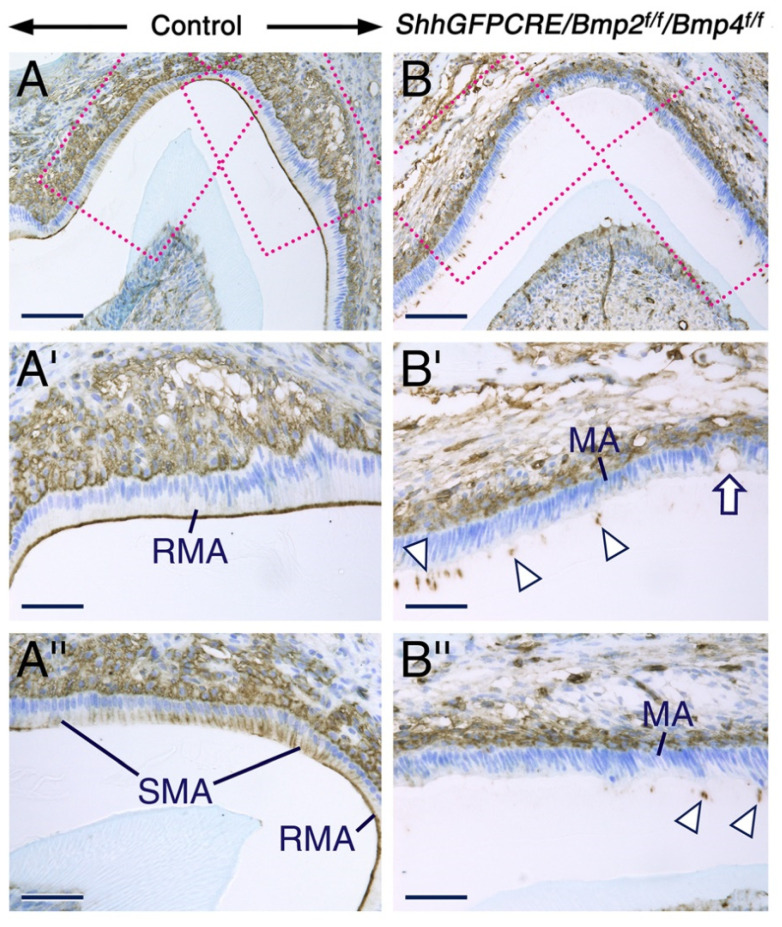
BMP2- and BMP4-deficient maturation-stage ameloblasts show cell-matrix and cell-cell contact anomalies and fail to exhibit a ruffled apical plasma membrane. (**A**,**B**) Representative sections across molars from 12 days post-partum control (**A**) and *ShhGFPCRE/Bmp2^f/f^/Bmp4^f/f^* mutant (**B**) mice after immunostaining (brown) for anti-phosphorylated ezrin/radixin/moesin (P-ERM). (**A’**,**B’**,**A’’**,**B’’**) are magnified views of the boxed areas in (**A**,**B**). In control teeth, P-ERM immunostaining decorates the ruffled apical plasma membrane of ruffle-ended maturation-stage ameloblasts (RMA) and is concentrated in the lateral membranes (at cell-cell adhesion contacts) of smooth-ended maturation-stage ameloblasts (SMA). By contrast, in mutant teeth, P-ERM is present only in cellular fragments embedded in the retained enamel matrix (Arrowheads in (**B’**,**B’’**)) and around microcysts within the ameloblast layer (arrow in (**B’**)), indicating failure of development of the ruffled apical plasma membrane and abnormal cell-cell contacts and cell-extracellular matrix attachment. Note the disorganization and abnormal attachment of the apical membrane of the mutant maturation-stage ameloblasts to the enamel matrix (**B**,**B’**,**B’’**). MA, maturation-stage ameloblasts; RMA, ruffle-ended maturation-stage ameloblasts; SMA, smooth-ended maturation-stage ameloblasts. Scale bars: 100 µm (**A**,**B**) and 50 µm (**A’**,**B’**,**A’’**,**B’’**).

To further characterize the phenotype of the mutant maturation-stage ameloblasts and to confirm that these cells fail to form a ruffed apical plasma membrane, as suggested by histology and anti-amelogenin staining, we used immunohistochemistry to detect phosphorylated (activated) ERMs (P-ERM). This revealed striking differences between the mutant and control teeth. In the control teeth, P-ERM immunostaining was concentrated in the ruffled apical membrane and in the lateral membranes of ruffle-ended and smooth-ended maturation-stage ameloblasts, respectively (Figure 3A,A’,A’’). By contrast in the mutant teeth, P-ERM immunoreactivity was undetectable in the apical and lateral plasma membranes of maturation-stage ameloblasts (Figure 3B,B’,B’’). Rather, the P-ERM immunostaining was robust in cellular fragments embedded in the enamel matrix and was also detectable in membranes around what appears to be holes and microcyst-like structures between ameloblasts (Figure 3B’). Zonula occludens 1 (ZO1), a protein that localizes at cell-cell contacts such as tight junctions [83,84], has been shown to be expressed in maturation-stage ameloblasts [85]. We found loss of ZO1 immunoreactivity between maturation-stage ameloblasts and the stratum intermedium at a site where subsets of maturation-stage ameloblasts appear to have begun disengagement from the ameloblast layer, while other maturation-stage ameloblasts were abnormally attached to the enamel matrix (Figure S5G,G’).

These data show that the mutant maturation-stage ameloblasts have altered cell-cell contacts, fail to develop a ruffled border, and display aberrant apical membranes that are abnormally attached to the enamel matrix. As the apical membranes of maturation-stage ameloblasts are the major site of reabsorption of EMP debris [53], with the ruffled border playing a prominent role in this process [21,86], these findings strongly suggest that the abnormal development of the apical membranes of mutant maturation-stage ameloblasts is the main cause of their failure to reabsorb EMP debris by endocytosis. Prior work used antibodies against ezrin, radixin and moesin that do not distinguish between the activated and non-activated forms of these proteins [82]. Our study thus shows that ERM that are activated through phosphorylation of threonine residues are involved in maturation-stage ameloblasts.

Taken together, these results demonstrate that combined BMP2 and BMP4 signaling in the dental epithelium is necessary for normal organization, morphological differentiation and function of maturation-stage ameloblasts, and for proper enamel formation. That the developmental aberrations of enamel and maturation-stage ameloblasts occur in both the *ShhGFPCRE/Bmp2^f/f^/Bmp4^f/f^* and *ShhGFPCRE/Bmp2^+/f^/Bmp4^f/f^* mutant mice indicates that one functional allele of *Bmp2* is insufficient to rescue the phenotype.

### 2.3. Altered Alkaline Phosphatase Activity in Maturation Stage Ameloblasts upon Combined Loss of Epithelial BMP2 and BMP4 Signaling

Tissue non-specific alkaline phosphatase (TNAP) is crucial for proper bone mineralization [87,88,89], and mice with loss-of-function of TNAP exhibit enamel defects [90,91,92,93]. BMPs, including BMP2 and BMP4, have been shown to stimulate TNAP enzymatic activity in cell cultures [60,94]. Previous studies have shown that in the dental epithelium the expression of TNAP mRNA and protein as well as TNAP activity are enriched in cells of the stratum intermedium during the secretory stage of amelogenesis [92,95,96,97,98,99,100], and that during the maturation stage TNAP activity is strong in the apical convoluted plasma membrane of ruffle-ended maturation-stage ameloblasts [86,97,98]. Furthermore, intracytoplasmic granules/vesicles with strong TNAP activity have been described in both smooth-ended and ruffle-ended maturation stage ameloblasts [97].

**Figure 4:**
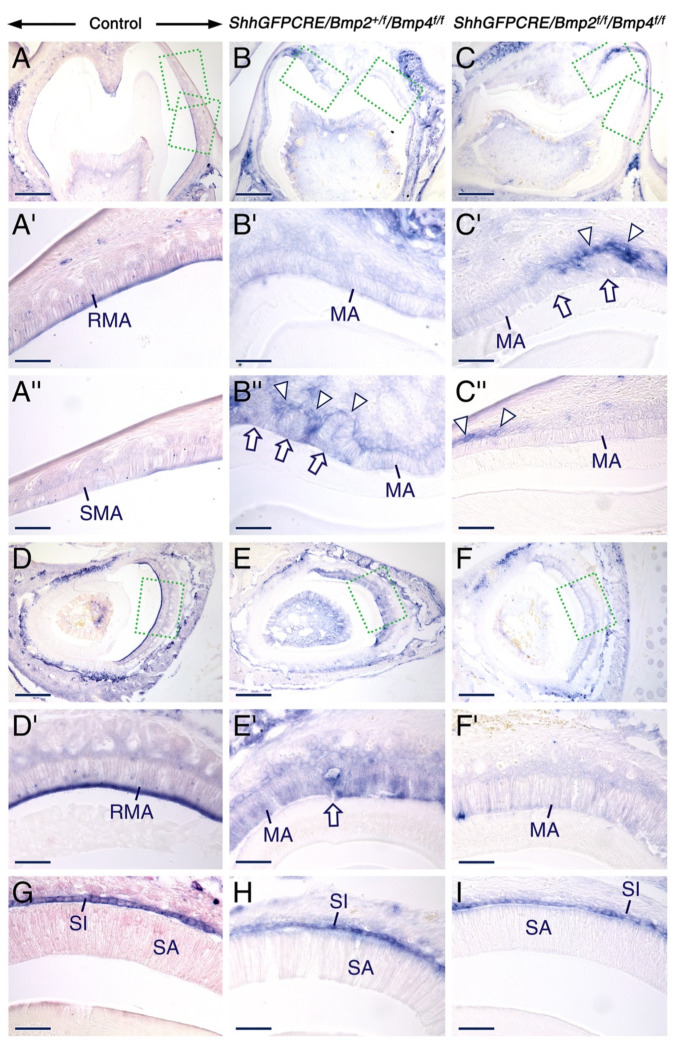
Combined loss of BMP2 and BMP4 signaling in the dental epithelium affects alkaline phosphatase activity at the maturation stage of amelogenesis. (**A**–**I**) Representative alkaline phosphatase histochemistry data in cryostat sections across molars (**A**–**C**) and incisors (**D**–**I**) from 12 days post-partum control (**A**,**D**,**G**), *ShhGFPCRE/Bmp2^+/f^/Bmp4^f/f^* (**B**,**E**,**H**), and *ShhGFPCRE/Bmp2^f/f^/Bmp4^f/f^* (**C**,**F**,**I**) mice showing the distribution of alkaline phosphatase activity (dark blue). (**A’**–**C’**) and (**A’’**–**C’’**) are magnified views of the boxed areas in (**A**–**C**). (**D’**–**F’**) are magnified images of the boxed areas in (**D**–**F**). In control teeth, alkaline phosphatase activity is strong in the apical border of ruffle-ended maturation-stage ameloblasts (RMA; (**A’**,**D’**)) as well as in intracytoplasmic vesicles in RMA (**A’**,**D’**) and smooth-ended maturation-stage ameloblasts (SMA; (**A’’**)). Severely decreased alkaline phosphatase activity in the *ShhGFPCRE/Bmp2^+/f^/Bmp4^f/f^* maturation-stage ameloblasts (**B’**,**B’’**,**E’**). Barely detectable alkaline phosphatase activity in the *ShhGFPCRE/Bmp2^f/f^/Bmp4^f/f^* maturation-stage ameloblasts (**C’**,**C’’**,**F’**). The arrowheads in (**B’’**,**C’**,**C’’**) indicate localized strong alkaline phosphatase activity in cells adjacent to maturation-stage ameloblasts. The arrows in (**B’’**,**C’**,**E’**) show sites where maturation-stage ameloblasts disengage from the ameloblast layer. At the secretory stage of amelogenesis, alkaline phosphatase activity in the stratum intermedium adjacent to secretory-stage ameloblasts is not altered in the mutant teeth (**G**–**I**). MA, maturation-stage ameloblasts; RMA, ruffle-ended maturation-stage ameloblasts; SA, secretory ameloblasts; SI, stratum intermedium; SMA, smooth-ended maturation-stage ameloblasts. Scale bars: 200 µm (**A**–**F**) and 50 µm (**A’**–**C’’**,**D’**–**I**).

To determine whether TNAP activity is defective in the *ShhGFPCRE/Bmp2^f/f^/Bmp4^f/f^* and *ShhGFPCRE/Bmp2^+/f^/Bmp4^f/f^* mutant ameloblasts, we carried out TNAP histochemistry. Consistent with previous findings [97], during the maturation stage of amelogenesis TNAP activity in control teeth was concentrated in the apical pole of the ruffle-ended ameloblasts in molars (Figure 4A,A’) and incisors (Figure 4D,D’) as well as in intracellular vesicles in both ruffle-ended (Figure 4A’,D’) and smooth-ended (Figure 4A’’) ameloblast. By contrast, in the *ShhGFPCRE/Bmp2^f/f^/Bmp4^f/f^* mutants, TNAP activity in maturation stage ameloblasts was nearly abrogated in molars (Figure 4C–C’’) and severely reduced in incisors (Figure 4F,F’). In the *ShhGFPCRE/Bmp2^+/f^/Bmp4^f/f^* mutants, while maturation stage ameloblasts in molars (Figure 4B–B’’) and incisors (Figure 4E,E’) exhibited weak but detectable TNAP activity, they lacked the distinct concentration of this enzyme’s activity to the apical pole of maturation-stage ameloblasts, and failed to show intracytoplasmic vesicles with TNAP activity that are as prominent as those observed within maturation-stage ameloblasts of control teeth. At the secretory stage, the mutant teeth exhibited normal TNAP activity which, at this stage and within the dental epithelium, is found in cells of the stratum intermedium (Figure 4G–I).

These data show that normal TNAP activity in maturation-stage ameloblasts requires combined BMP2 and BMP4 signaling. Furthermore, these data provide further evidence of aberrant apical membrane of the mutant maturation-stage ameloblasts.

### 2.4. Normal Gene Expression Patterns in Secretory and Maturation-Stage Ameloblasts and Unaltered KLK4 Secretion upon Combined Loss of Epithelial BMP2 and BMP4 Signaling

**Figure 5:**
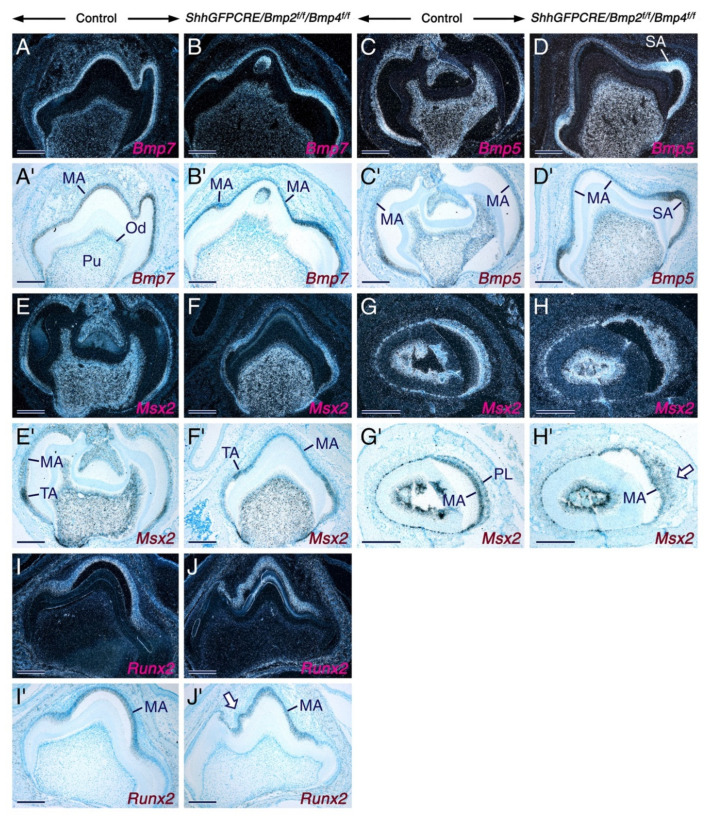
Normal expression patterns of *Bmp5*, *Bmp7*, *Msx2,* and *Runx2* upon combined loss of BMP2 and BMP4 signaling in the dental epithelium. (**A**–**J’**) Representative in situ hybridization data in sections across molars (**A**–**F’**,**I–J’**) and incisors (**G**–**H’**) from 12 days post-partum control and *ShhGFPCRE/Bmp2^f/f^/Bmp4^f/f^* mice showing the expression patterns of *Bmp7* (**A**–**B’**), *Bmp5* (**C**–**D’**), *Msx2* (**E**–**H’**), and *Runx2* (**I**–**J’**). The hybridization signals appear as shiny or black dots in dark-field (**A**–**J**) and bright-field (**A’**–**J’**) images, respectively. In the mutant teeth, the intensity of *Bmp5*, *Bmp7*, *Msx2,* and *Runx2* hybridization signals in maturation-stage ameloblasts, including in cells that have migrated away from the ameloblast layer (arrow in (**H’**)) and in cells within regions of disrupted ameloblast layer (arrow in (**J’**)), is indistinguishable from that in maturation-stage ameloblasts of control teeth. MA, maturation-stage ameloblasts; Od, odontoblasts; Pl, papillary layer; SA, late secretory ameloblasts; TA, transition-stage ameloblasts; Pu, dental pulp tissue. Scale bars: 200 µm (**A**–**J’**).

**Figure 6:**
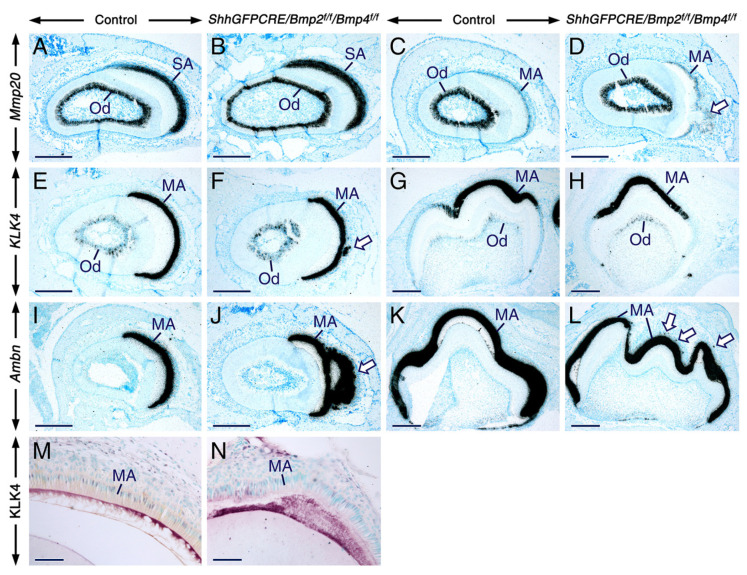
Normal expression patterns of *Mmp20*, *Klk4*, and *ameloblastin* and normal distribution of KLK4 upon combined loss of BMP2 and BMP4 signaling in the dental epithelium. (**A**–**L**) Representative bright-field images of sections across teeth from 12 days post-partum control (**A**,**C**,**E**,**G**,**I**,**K**) and *ShhGFPCRE/Bmp2^f/f^/Bmp4^f/f^* mutant (**B**,**D**,**F**,**H**,**J**,**L**) mice showing the expression patterns of *Mmp20* (**A**–**D**), *Klk4* (**E**–**H**), and *ameloblastin* (*Ambn;* (**I**–**L**)) visualized by in situ hybridization. Sections across incisors at the level of the secretory stage of amelogenesis (**A**,**B**). Sections across incisors (**C**–**F**,**I**,**J**) and molars (**G**,**H**,**K**,**L**) at the level of the maturation stage of amelogenesis. The intensity of hybridization signals (black dots) for *Mmp20*, *Klk4*, and *Ambn* in the mutant ameloblasts is unaffected, and in both control and mutant teeth maturation-stage ameloblasts express low levels of *Mmp20* and high levels of *Klk4* and *Ambn*. Arrows in (**D**,**F**) and (**J**) indicate mutant maturation-stage ameloblasts that undergo collective migration away from the ameloblast layer. Arrows in (**L**) show mutant maturation-stage ameloblasts that quit the ameloblast layer as single cells. (**M**,**N**) Immunohistochemistry showing the distribution of kallikrein 4 (KLK4) protein (red) in sections across incisors from adult control (**M**) and *ShhGFPCRE/Bmp2^f/f^/Bmp4^f/f^* mutant (**N**) mice at the level of early maturation stage. Similar to the control incisor, the mutant incisor shows KLK4 immunoreactivity in the enamel extracellular matrix, indicating that the secretory function of the mutant maturation-stage ameloblasts is unaltered. MA, maturation-stage ameloblasts; Od, odontoblasts; SA, secretory ameloblasts. Scale bars: 200 µm (**A**–**L**) and 50 µm (**M**,**N**).

To assess further the impact of loss of BMP2 and BMP4 signaling on ameloblasts, we used in situ hybridization to analyze the expression patterns of genes known to be expressed in secretory ameloblasts (*ameloblastin*, *Bmp5*, *Bmp7*, *Mmp20,* and *Msx2*) and/or maturation stage ameloblasts (*ameloblastin*, *Bmp5*, *Bmp7*, *Klk4*, *Mmp20*, *Msx2*, and *Runx2*). However, we found that compared to the control ameloblasts, the mutant ameloblasts did not display altered levels of hybridization signals for these genes (Figure 5 and Figure 6). These data demonstrate that, despite their failure to develop the characteristic morphological features of maturation-stage ameloblasts, the mutant maturation-stage ameloblasts retained the ability to express genes known to be activated in this cell type.

In the mutant teeth, the expression patterns of *Runx2* (Figure 5J,J’), *Mmp20* (Figure 6D)**,** *Klk4* (Figure 6F), and *ameloblastin* (Figure 6J,L) provided further evidence that the cells that emigrate away from the ameloblast layer and form nodules were indeed maturation-stage ameloblasts. Ameloblastin in situ hybridization also revealed that subsets of maturation-stage ameloblasts emigrate from the ameloblast layer as single cells (Figure 6L). The fact that the mutant maturation-stage ameloblasts are abnormal despite normally expressing *Bmp5* and *Bmp7* demonstrates that BMP2 and BMP4 are absolutely required for proper morphological differentiation and function of these cells, and that BMP2 and BMP4 activities are not totally compensated by the activities of BMP5 and BMP7.

Immunostaining for KLK4 showed that, despite their aberrant morphology, the mutant maturation-stage ameloblasts are able to secrete KLK4 into the enamel matrix (Figure 6M,N). This, together with the finding that the mutant maturation-stage ameloblasts secrete ameloblastin protein as shown in Figure 2, suggests that the secretory activity of these cells is not affected.

### 2.5. Combined Loss of BMP2 and BMP4 Signaling in the Dental Epithelium Causes Massive Apoptosis in the Stratum Intermedium

**Figure 7:**
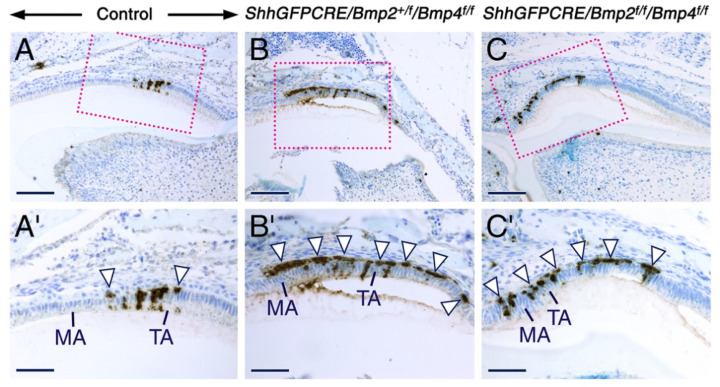
Combined loss of BMP2 and BMP4 signaling in the dental epithelium causes massive apoptosis in the stratum intermedium. (**A**–**C**) Representative immunostaining for cleaved lamin (**A**) showing the distribution of apoptotic cells (brown) in sections across molars from 12 days post-partum control (**A**), *ShhGFPCRE/Bmp2^+/f^/Bmp4^f/f^* (**B**), and *ShhGFPCRE/Bmp2^f/f^/Bmp4^f/f^* (**C**) mice. (**A’**–**C’**) are magnified views of the boxed areas in (**A**–**C**). In the control teeth, only a few apoptotic cells are detectable in the stratum intermedium adjacent to transition-stage ameloblasts (arrowheads in (**A’**)). By contrast, in the mutant teeth, cells of the stratum intermedium adjacent to both the transition-stage and early maturation-stage ameloblasts show massive apoptosis (arrowheads in (**B’**,**C’**)). MA, early maturation-stage ameloblasts; TA, transition-stage ameloblasts. Scale bars: 100 µm (**A**–**C**) and 50 µm (**A’**–**C’**).

BMP signaling regulates cell proliferation, cell survival, and apoptosis in a context-dependent manner [60,101,102,103,104]. To determine whether loss of BMP2 and BMP4 signaling has a deleterious effect on the survival of maturation-stage ameloblasts, we carried out immunostaining for cleaved lamin A to detect apoptotic cells. In the control teeth, apoptosis was expectedly detectable in subsets of transition-stage ameloblasts and in some cells of the stratum intermedium overlying transition-stage ameloblasts (Figure 7A,A’). However, in the mutant teeth, apoptotic figures were readily detectable not only in subsets of transition-stage ameloblasts, but also in some early maturation-stage ameloblasts (Figure 7B–C’). Strikingly, within the stratum intermedium, the area with apoptotic figures was expanded and covered stratum intermedium cells adjacent to transition-stage and early maturation-stage ameloblasts, indicating massive apoptosis in this cell layer (Figure 7B–C’). These findings suggest that combined BMP2 and BMP4 signaling is crucial for survival of the stratum intermedium during the transition and early maturation stages of amelogenesis.

## 3. Discussion

### 3.1. BMP2 and BMP4 Signaling in the Dental Epithelium Is not Required for Early Stages of Tooth Development

Studies of mouse models revealed key roles for BMP signaling during tooth formation. Epithelial or mesenchymal loss of the gene encoding type 1 BMP receptor1a (Bmpr1a) cause tooth developmental arrest [105,106], and deactivation of the Bmp4 gene in the dental mesenchyme leads to aberrant tooth formation [107]. Deletion of the *Bmpr1a* gene in odontoblasts [108] and single or combined loss of *Bmp2* and *Bmp4* gene function in the odontoblast lineage result in abnormal dentin formation [109,110,111]. *K14-CRE/Bmp2^f/f^/Bmp4^f/f^* mice with disabled *Bmp2* and *Bmp4* genes in the dental epithelium display abnormal enamel maturation [58], and mice overexpressing the BMP inhibitor noggin in the dental epithelium exhibit an array of tooth anomalies, including enamel defects [112,113].

In this study, we used the *ShhGFPCRE* knock-in allele, which enables deactivation of *Bmp2* and *Bmp4* from early stages of odontogenesis onwards in dental epithelial cells that express *Shh* and their descendants. However, and consistent with previous finding in *K14-CRE/Bmp2^f/f^/Bmp4^f/f^* mutant mice [58], in the *ShhGFPCRE/Bmp2^f/f^/Bmp4^f/f^* and *ShhGFPCRE/Bmp2^+/f^/Bmp4^f/f^* mutants, early stages of tooth formation proceeded normally, and at the secretory stage of enamel formation the mutant teeth were undistinguishable from the control teeth.

Previous studies have shown that differentiation of preameloblasts into secretory ameloblasts is induced by and requires BMP signals emanating from the dental papilla mesenchyme and odontoblasts [57,114]. These BMP signals reach preameloblasts as these cells are not separated from the dental papilla mesenchyme and odontoblasts by thick layers of extracellular matrices during early stages of tooth formation. Besides expressing *Bmp2* and *Bmp4*, the enamel knots and the ameloblast lineage, including secretory ameloblasts, also express *Bmp7* [57,63]. In addition, secretory ameloblasts express high levels of *Bmp5* [63]. It is thus possible that BMP signaling emanating from the dental mesenchyme and/or BMP5 and BMP7 signaling within the dental epithelium may have prevented putative deleterious effects of loss of BMP2 and BMP4 in the dental epithelium, thus enabling the *ShhGFPCRE/Bmp2^f/f^/Bmp4^f/f^* and *ShhGFPCRE/Bmp2^+/f^/Bmp4^f/f^* mutant teeth to undergo normal morphogenesis and to develop normal secretory ameloblasts.

### 3.2. Maturation-Stage Ameloblast Morphological Differentiation and Function Depend on Epithelial BMP2 and BMP4 Inputs to Ensure Proper Enamel Maturation

Similar to previous findings in *K14-CRE/Bmp2^f/f^/Bmp4^f/f^* mutant mice [58], we found that loss of BMP2 and BMP4 signaling in the *ShhGFPCRE/Bmp2^f/f^/Bmp4^f/f^* and *ShhGFPCRE/Bmp2^+/f^/Bmp4^f/f^* mutants leads to abnormal enamel maturation characterized by retention of enamel matrix containing enamel matrix proteins (EMPs), a defect that characterizes enamel in a condition known as hypomaturation amelogenesis imperfecta.

Our study further revealed that epithelial BMP2 and BMP4 signaling is crucial for proper morphological differentiation and function of maturation-stage ameloblasts, as well as for their normal organization within the maturation-stage ameloblast layer. Indeed, combined loss of epithelial BMP2 and BMP4 inputs in the *ShhGFPCRE/Bmp2^f/f^/Bmp4^f/f^* and *ShhGFPCRE/Bmp2^+/f^/Bmp4^f/f^* mutant teeth caused development of severely dysmorphic maturation-stage ameloblasts with abnormal cell-cell contacts and cell-matrix adhesion. These cells also failed to exhibit a ruffled apical plasma membrane and to reabsorb EMPs. Remarkably, mutant maturation-stage ameloblasts also underwent pathological emigration away from the ameloblast layer. By contrast, no histopathological alterations of maturation-stage ameloblasts have been reported in the *K14-CRE/Bmp2^f/f^/Bmp4^f/f^* mutant teeth studied previously [58]. In the present work, we showed that at the maturation stage of amelogenesis, teeth from *K14-CRE/R26R* and *ShhGFPCRE/R26R* reporter mice exhibited similar distribution patterns of CRE activity in the dental epithelium, including in maturation-stage ameloblasts. Therefore, *K14-CRE*- and *ShhGFPCRE*-mediated deactivation of *Bmp2* and *Bmp4* gene function are expected to cause similar, if not identical, structural and functional defects in maturation-stage ameloblasts. Our findings thus suggest that the phenotype of *K14-CRE/Bmp2^f/f^/Bmp4^f/f^* maturation-stage ameloblasts might have been overlooked in the previous study [58]. Indeed, this was the case, as upon examination of the figures published previously [58], we noticed that the *K14-CRE/Bmp2^f/f^/Bmp4^f/f^* mutant maturation-stage ameloblasts display morphological defects similar to those we have uncovered in the *ShhGFPCRE/Bmp2^f/f^/Bmp4^f/f^* and *ShhGFPCRE/Bmp2^+/f^/Bmp4^f/f^* mutant teeth.

Enamel maturation into a hard tissue requires degradation and nearly total removal of EMPs from the enamel matrix [8,115]. While MMP20 is essential for processing EMPs into small peptides, KLK4 is involved in their degradation into even smaller fragments [8,44,50], thus facilitating their removal from the maturing enamel by endocytosis [53]. In the *K14-CRE/Bmp2^f/f^/Bmp4^f/f^* mutant teeth, the expression of *Mmp20* and *Klk4* has been reported to be downregulated at the maturation stage [58]. By contrast, we found no alterations in the expression of *Mmp20* and *Klk4* in the *ShhGFPCRE/Bmp2^f/f^/Bmp4^f/f^* maturation-stage ameloblasts. These differences could be due to the different in situ hybridization (ISH) techniques used, as in the previous study [58] a chromogenic ISH procedure was used, whereas in the present study we used the more sensitive radioactive ISH.

Our findings thus suggest that aberrant development and function of maturation-stage ameloblasts is the major cause for defective enamel maturation in the *ShhGFPCRE/Bmp2^f/f^/Bmp4^f/f^* and *ShhGFPCRE/Bmp2^+/f^/Bmp4^f/f^* mutants.

### 3.3. Defective Resorptive Activities of Bmp2/Bmp4-Deficient Maturation-Stage Ameloblasts Is Likely a Cause for Failure of Proper Enamel Maturation

Currently, it is believed that maturation-stage ameloblasts remove EMP degradation products from the maturing enamel through endocytosis [52,53], and that the ruffle-ended ameloblasts play a major role in absorption of extracellular molecules through their apical ruffled border [21,86]. The smooth-ended maturation-stage ameloblasts have been suggested to be involved in basal transport activities [86]. We found that, unlike maturation-stage ameloblasts in control teeth which exhibited a distinct apical ruffled border, as evidenced by histology, tissue non-specific alkaline phosphatase (TNAP) activity as well as by immunostaining for amelogenin and phosphorylated ezrin/radixin/moesin (P-ERM), maturation-stage ameloblasts in the mutant teeth not only failed to display these features, but they also exhibited apical membranes aberrantly attached to the enamel matrix. In addition, despite showing LAMP1-positive vesicles, the mutant maturation-stage ameloblasts did not exhibit any amelogenin-positive intracellular vesicles. Furthermore, at the maturation stage, the mutant teeth showed immunostaining for amelogenin and ameloblastin in the abnormally retained enamel matrix.

These data strongly suggest that the defective endocytotic activity of mutant maturation-stage ameloblasts is caused by abnormal development of their apical membranes. This led to accumulation of EMP degradation products in the enamel matrix, eventually leading to abnormal enamel maturation.

Taken together, our findings show that proper morphological differentiation and normal function of maturation-stage ameloblasts to ensure normal enamel maturation crucially requires combined BMP2 and BMP4 signaling.

TNAP is a membrane-bound enzyme that may play a role in tissue mineralization through hydrolysis of inorganic pyrophosphate, a known inhibitor of hydroxyapatite crystal development [116,117], and has been suggested to be involved in other cellular functions, including transmembrane movement of ions and metabolites [118,119,120] and cell adhesion [121].

We found that TNAP activity in maturation-stage ameloblasts was nearly abrogated and severely decreased in the *ShhGFPCRE/Bmp2^f/f^/Bmp4^f/f^* and *ShhGFPCRE/Bmp2^+/f^/Bmp4^f/f^* mutant teeth, respectively. In cell cultures, BMP2 and BMP4 have been shown to induce TNAP activity [60,94]. Our data thus provide strong in vivo evidence for the dependence of TNAP activity on BMP signaling.

We also found that, in the *ShhGFPCRE/Bmp2^+/f^/Bmp4^f/f^* teeth, maturation-stage ameloblasts failed to exhibit concentration of TNAP activity in their apical membrane and lacked prominent intracellular vesicles with TNAP activity. The exact function of TNAP in maturation-stage ameloblasts is still unknown. It has been reported that increased mineralization of enamel correlates well with the enrichment of TNAP activity in the convoluted apical membrane of ruffle-ended maturation-stage ameloblasts [97], and that the presence of TNAP activity in intracellular vesicles within maturation-stage ameloblasts suggests that these cells are involved in membrane endocytosis [97]. Taken together with our findings, these observations further support the likelihood of abnormal endocytotic activity of maturation-stage ameloblasts upon loss of BMP2 and BMP4 signaling.

Mutations of the human gene encoding TNAP cause hypophosphatasia, a condition characterized by defective mineralization of the skeleton and tooth anomalies, including premature loss of teeth and enamel hypoplasia [122,123,124]. Mice with deactivation of *Alpl*, the TNAP-encoding gene, exhibit dental aberrations, including enamel defects [90,91,92] and development of dysmorphic and disorganized maturation-stage ameloblasts [92].

In light of these findings, we propose that defective TNAP activity may also be a causal factor for the abnormal development and defective function of maturation-stage ameloblasts that are deprived of BMP2 and BMP4 signaling.

### 3.4. Signaling from BMP2 and BMP4 in the Dental Epithelium Is Required for Survival of the Stratum Intermedium and for Normal Organization of Maturation-Stage Ameloblasts

At the maturation stage of amelogenesis, the retained enamel matrix in the *ShhGFPCRE/Bmp2^f/f^/Bmp4^f/f^* and *ShhGFPCRE/Bmp2^+/f^/Bmp4^f/f^* mutant teeth was wavy, and in each mutant tooth, subsets of mutant maturation-stage ameloblasts underwent single or collective emigration from the ameloblast layer and formed, together with other epithelial cells of the enamel organ, prominent tumor-like nodules, some of which encompassed enamel matrix-like material. Disorganization of early maturation-stage ameloblasts and formation of multicellular masses encompassing ectopic enamel matrix-like substance have been reported to occur in mice lacking the function of MMP20 which also exhibit severe enamel hypoplasia and abnormal secretory ameloblasts [39,40,41,50]. In other mouse models, such as mice carrying alterations in the genes encoding amelogenin [26,125] and ameloblastin [42,45], ameloblasts are already overtly disorganized at the secretory stage, and abnormal multicellular masses adjacent to extracellular matrix become prominent at the maturation stage of amelogenesis.

What causes maturation-stage ameloblasts to delaminate and emigrate away from the cell layer? In the *ShhGFPCRE/Bmp2^f/f^/Bmp4^f/f^* and *ShhGFPCRE/Bmp2^+/f^/Bmp4^f/f^* mutant teeth one possible trigger of extrusion of maturation-stage ameloblasts could be their aberrant morphological differentiation, which may lead to or be a result of aberrant cell-cell contacts and/or cell-matrix adhesion, as shown, for example, by P-ERM immunostaining. Consistent with this notion, in *Drosophila* wing disc epithelium, decapentaplegic/BMP (Dpp/BMP) signaling has been shown to be essential for normal epithelial cell morphology and integrity and to play a key role in regulating the organization of the cytoskeleton, as cells with loss of Dpp/BMP signaling, are severely misshapen and extrude from the tissue layer as viable cysts [126,127].

Our study also revealed that, in control teeth, cells of the stratum intermedium express *Bmp2* and *Bmp4*, and that this cell layer underwent CRE-mediated ablation of *Bmp2* and *Bmp4* in the mutant teeth. We showed that the mutant teeth exhibited massive apoptosis in cells of the stratum intermedium adjacent to transition-stage and early maturation-stage ameloblasts, but ameloblast migration was not readily observed at the transition and early maturation stages of amelogenesis. Then later, after clearance of apoptotic bodies in the stratum intermedium (early maturation stage and maturation stage), it seemed that subsets of mutant maturation-stage ameloblasts underwent migration away from the ameloblast layer. Furthermore, we observed a nested loss of ZO-1, a cell junctional protein, at the basal pole (between ameloblasts and the stratum intermedium) of mutant transition-stage ameloblasts that are likely beginning to emigrate from the ameloblast layer.

During enamel formation, the stratum intermedium forms a layer adjacent to ameloblasts [2]. During the transition-stage of amelogenesis, subsets of cells of the stratum intermedium and as many as 25% transition-stage ameloblasts normally perish by apoptosis [2,7,19,20,128]. Morphometric analyses of the enamel organ have shown that each stratum intermedium cell may form connections with 2–3 ameloblasts within the same ameloblast row and with at least two ameloblasts in adjacent rows [15]. It has been suggested that the stratum intermedium stabilizes differentiating ameloblasts [129] and is involved in co-ordinating the movement of ameloblasts during enamel formation [15]. It is also believed that the stratum intermedium/papillary layer produce signals to maintain maturation-stage ameloblast function [130]. Genetic studies in mice have shown that defective development of the stratum intermedium is associated with abnormal differentiation of ameloblasts [55,131], and that pathological separation of the stratum intermedium/papillary layer from ameloblasts severely impacts upon the morphology and function of maturation-stage ameloblasts [130,132].

In light of these observations and our findings, it is possible to surmise, that in the *ShhGFPCRE/Bmp2^f/f^/Bmp4^f/f^* and *ShhGFPCRE/Bmp2^+/f^/Bmp4^f/f^* mutant teeth, disruption of the stratum intermedium by massive apoptosis had deleterious effects upon the cytodifferentiation process and organization of maturation-stage ameloblasts. This may have contributed to the phenotypic changes in mutant maturation-stage ameloblasts, including their exodus from the ameloblast tissue layer.

## 4. Materials and Methods

### 4.1. Ethics Statement

Mouse research studies were reviewed and approved by the Animal Research Ethics Committee in Göteborg, Sweden [Dnr. 230-2010 (29 September 2010), Dnr. 174-2013 (12 November 2013), Dnr. 40-2016 (27 April 2016), and Dnr. 5.8.18-15468/2018 (5 December 2018)].

### 4.2. Mouse Lines

The CRE-driver mouse lines used in this study are the *ShhGFPCRE* knock-in mice [133] and *keratin14-CRE* (*K14-CRE*) transgenic mice [54]. Mice with floxed (f) alleles of the *Bmp2* (*Bmp2^f/f^* [134,135]) and *Bmp4* (*Bmp4^f/f^* [134,136,137]) genes were used to induce ShhGFPCRE–mediated deactivation of these genes in the dental epithelium. To generate *ShhGFPCRE/Bmp2^f/f^/Bmp4^f/f^* and *ShhGFPCRE/Bmp2^+/f^/Bmp4^f/f^* double mutants (“+” indicates the wild-type allele) we crossed heterozygous *ShhGFPCRE/Bmp2^+/f^/Bmp4^+/f^* males with *Bmp2^f/f^/Bmp4^f/f^* females. *K14-CRE/R26R* and *ShhGFPCRE/R26R* mice carrying the *R26R* reporter allele [138] were generated as described previously [55,74,139]. The mice were genotyped by polymerase chain reaction analyses as described previously [54,133,134]. Mice without the CRE or floxed alleles were phenotypically normal; they were thus used as controls.

### 4.3. Histology, Immunohistochemistry and In Situ Hybridization

Jaws from control and mutant mice at different postnatal developmental stages (from post-partum day 1 to adulthood), were prepared and processed for paraffin embedding as described previously [74,140]. Briefly, the jaws were fixed overnight at 4 °C in either 4% paraformaldehyde (PFA) in phosphate buffered saline (PFA/PBS) or in 95% ethanol containing 1% glacial acetic acid (ethanol-acetic acid). The jaws were subsequently demineralized as described previously [74].

Six-µm-thick dewaxed tissue sections from ethanol-acetic acid-fixed specimens were used for histological staining with Alcian blue van Gieson and for immunohistochemistry. For immunohistochemical visualization of apoptotic cells, sections from PFA/PBS-fixed specimens were used. Immunostaining was carried out as described previously [74,140].

Rabbit polyclonal anti-amelogenin (1:3000 dilution) was obtained from Kamiya Biomedical Company (Seattle, WA, USA). Rabbit polyclonal anti-carbonic anhydrase II (1:1000 dilution), rabbit polyclonal anti-carbonic anhydrase VI (1:3000 dilution), Rabbit polyclonal anti-Bax (P-19; 1:2000 dilution), goat anti-ameloblastin (N-18; 1:4000 dilution) and rat monoclonal anti-LAMP1 (Clone 1D4B; 1:40,000 dilution) were from Santa Cruz Biotechnology (Dallas, TX, USA). Rabbit anti-ZO1 (1:500) was from Zymed Laboratories (South San Francisco, CA, USA). Rabbit anti-kallikrein 4 (dilution 1:2000) was a generous gift from Dr. J. Simmer. Rabbit anti-phospho-ezrin (Thr567)/radixin (Thr564)/moesin (Thr558) (dilution 1:200) and rabbit polyclonal anti-cleaved lamin A (small subunit; 1:1000 dilution) were from Cell Signaling Technology (Danvers, MA, USA).

Six-µm-thick dewaxed sections from PFA/PBS-fixed specimens were processed for in situ hybridization using either oligonucleotide probes or ^35^S-UTP-labelled riboprobes as previously described [74,140,141]. In situ hybridization with the oligonucleotide probes Mm-*Bmp2* (NM_007553.3; target sequence: 1545–2322) and Mm-*Bmp4* (NM_007554.2; target sequence: 804–1633) targeting deleted sequences in the *Bmp2* and *Bmp4* genes, respectively, was carried out using the Advanced Cell Diagnostics RNAscope technology (Bio-Techne, Oxon, UK). The ^35^S-UTP-labelled riboprobes *amelin*/*ameloblastin* [55,142,143], *Bmp5* and *Bmp7* [55,63], *Msx2* [55], *Runx2* [144], as well as *Klk4* and *Mmp20* [11] were generated from linearized plasmids. Following in situ hybridization, the tissue sections were counterstained with Richardson’s azur II-methylene blue.

Sections across jaws/teeth from at least three [from 12 days post-partum (dpp) onwards] control, *ShhGFPCRE/Bmp2^f/f^/Bmp4^f/f^*, and *ShhGFPCRE/Bmp2^+/f^/Bmp4^f/f^* mice were used for Alcian blue van Gieson staining and immunohistochemistry. Tissue sections from a control mouse and a *ShhGFPCRE/Bmp2^f/f^/Bmp4^f/f^* mutant mouse were stained with antibodies against carbonic anhydrase II, carbonic anhydrase VI and kallikrein-4. Tissue sections from two control and two *ShhGFPCRE/Bmp2^f/f^/Bmp4^f/f^* mutant mice were processed for anti-phosphorylated ezrin/radixin/moesin. Alcian blue van Gieson staining of tooth sections at 1 dpp was carried out in specimens from a mouse of each genotype. For in situ hybridization with oligonucleotide probes, sections across jaws/teeth from two control and two mutant mice were studied. In situ hybridization with radiolabeled riboprobes was carried out mainly in control and *ShhGFPCRE/Bmp2^f/f^/Bmp4^f/f^* mutant mice, and sections across jaws/teeth from at least two mice of each genotype were analyzed.

### 4.4. β-Galactosidase Histochemistry

For detection of CRE activity we carried out β-galactosidase histochemistry. Jaws from *K14-CRE/R26R* and *ShhGFPCRE/R26R* reporter mice were fixed overnight at 4 °C in 2% PFA/PBS and subsequently demineralized in 10% EDTA, pH 7.3. After 4–5 weeks, the decalcified specimens were washed in PBS, cryo-protected in 30% sucrose in PBS, and embedded in OCT compound. Cryostat sections (12 µm-thick) across jaws/teeth were processed for β-galactosidase staining as described previously [145]. For visualization of CRE activity in reporter embryos (embryonic day (E)14.5 and E18.4) and perinatal pups (newborns and 3 dpp), heads or jaws were processed for whole-mount β-galactosidase staining, paraffin embedding and counterstaining of dewaxed tissue sections with nuclear fast red as described previously [145]. Tissues from a *ShhGFPCRE/R26R* mouse at each developmental stage and a *K14-CRE/R26R* mouse at 12 dpp were processed for β-galactosidase histochemistry.

### 4.5. Tissue Non-Specific Alkaline Phosphatase Activity

For detection of tissue non-specific alkaline phosphatase (TNAP) activity by histochemistry, jaws/teeth from a mouse of each genotype were fixed in neutral buffered formalin and demineralized in 10% EDTA, PH 7.3. After decalcification, the specimens were washed in PBS and embedded in OCT compound. Cryostat sections (12 µm-thick) were preincubated for 10 min in a buffer consisting of 100 mM NaCl, 50 mM MgCl_2_, 100 mM Tris-HCl, pH 9.5 and 0.1% Tween 20. Thereafter, sections were incubated in BM-purple alkaline phosphatase substrate buffer (Sigma Aldrich Sweden, Stockholm; currently available at MERCK, Darmstadt, Germany) at room temperature until formation of a chromogenic precipitate.

## 5. Conclusions

BMP signaling plays determinant roles in various biological processes by controlling events such as cell fate determination, cell proliferation, and survival, as well as cell differentiation [60,62]. The aim of this work was to decipher the role of BMP2 and BMP4 signaling in maturation-stage ameloblasts by using an in vivo genetic approach that disables both the *Bmp2* and *Bmp4* genes in the epithelium of mouse teeth. Our study revealed that proper morphological differentiation and function of maturation-stage ameloblasts as well as their organization crucially depend on inputs from BMP2 and BMP4 signaling. Upon combined loss of BMP2 and BMP4 activities, maturation-stage ameloblasts undergo severe morphological and behavioral changes and are unable to perform the task of ensuring proper enamel maturation by reabsorbing enamel matrix proteins. This causes development of immature enamel, leading to severe wear of the mutant teeth.

We also showed that cells of the stratum intermedium, which form a layer adjacent to ameloblasts, express *Bmp2* and *Bmp4* and that in the mutant teeth this cell layer undergoes massive apoptosis, indicating that combined BMP2 and BMP4 inputs are instrumental for the survival of the stratum intermedium at the transition/early maturation stages. This abnormal apoptosis, together with mutant maturation-stage ameloblasts exhibiting abnormal cell-cell adhesion and cell-matrix attachment, likely contributed to migration of subsets of maturation-stage ameloblasts away from the ameloblasts layer, forming tumor-like structures.

Our study thus shows how several biological processes necessary for normal development and function of maturation-stage ameloblasts crucially require combined BMP2 and BMP4 signaling.

## Supplementary Materials

The following supporting information can be downloaded at: https://www.mdpi.com/article/10.3390/ijms23116095/s1.

## Abbreviations

Ambn/*Ambn*	Ameloblastin protein/gene
AMEL/*AmelX*	Amelogenin protein/gene
BMP/*Bmp*	Bone morphogenetic protein/gene
CA	Carbonic anhydrase
CRE/*CRE*	(Cyclization recombination) DNA recombinase protein/gene
DPP/*dpp*	Drosophila decapentaplegic protein/gene
EMPs	Enamel matrix proteins
ERM	Ezrin/radixin/moesin
*f*	Floxed allele
KLK4/*Klk4*	Kallikrein-4 protein/gene
LAMP1	Lysosome-associated membrane protein
MMP20/Mmp20	Matrix metalloproteinase 20 protein/gene
*MSX2/Msx2*	Muscle segment homeobox 2 protein/gene
RMA	Ruffle-ended maturation-stage ameloblasts
*RUNX2/Runx2*	Runt-related transcription factor 2 protein/gene
SHH/*Shh*	Sonic Hedgehog protein/gene
SMA	Smooth-ended maturation-stage ameloblasts
TNAP/*Alpl*	Tissue non-specific alkaline phosphatase protein/gene
ZO1	Zonula occludens1
