# Supplementary material for: Loss of BMP2 and BMP4 Signaling in the Dental Epithelium Causes Defective Enamel Maturation and Aberrant Development of Ameloblasts
Source: Int J Mol Sci. 2022 May 29;23(11):6095. doi: 10.3390/ijms23116095 (PMC9180982; doi:10.3390/ijms23116095)
Supplement: Supplementary file 1 [file ijms-23-06095-s001.zip › ijms-1705014-supplementary.pdf]

Supplementary Figures

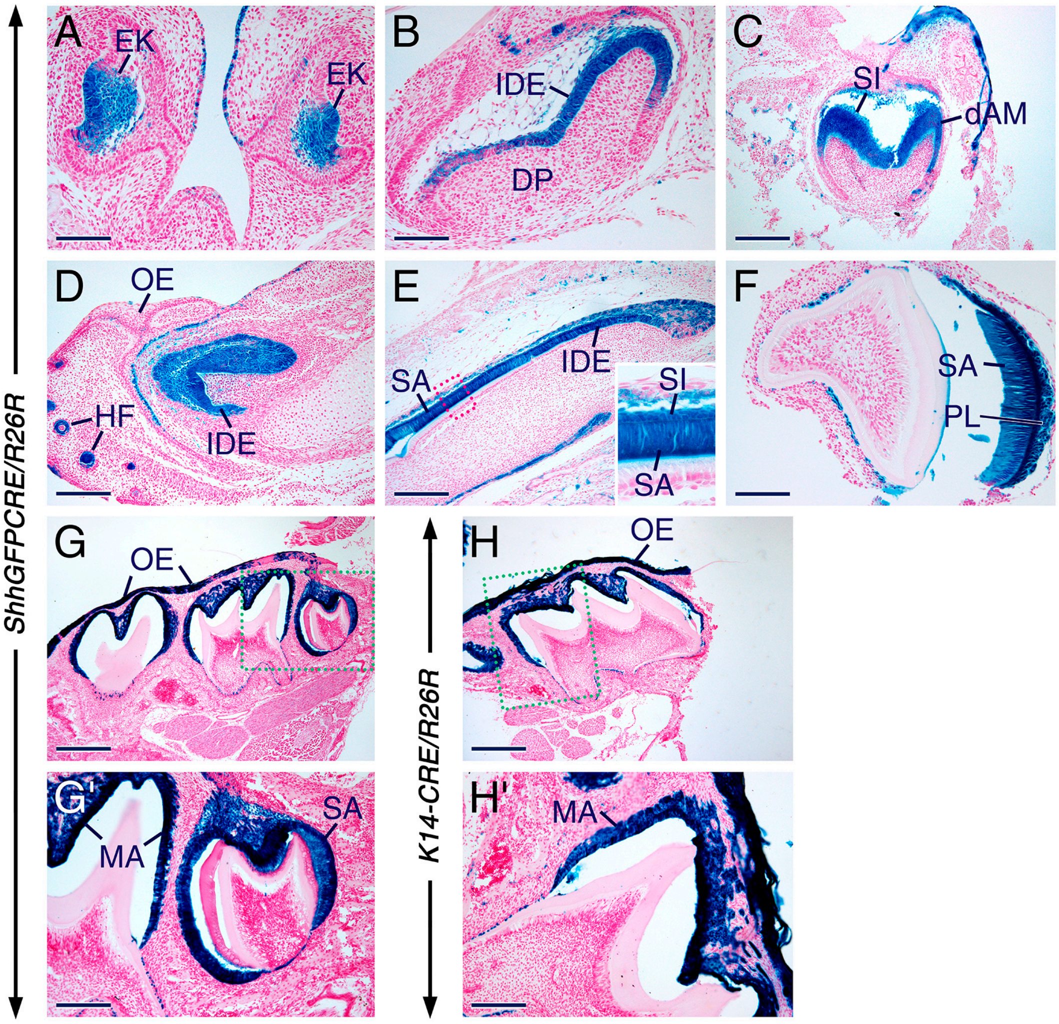

Figure S1. Distribution of ShhGFPCRE and K14-CRE activities during tooth development. **(A–H)** Representative β-galactosidase histochemistry showing the distribution of CRE activity (blue) in developing teeth from *ShhGFPCRE/R26R* (**A–G**) *and K14-CRE/R26R* (**H**) reporter mice. Frontal sections (**A–C**) across the first (**A**,**C**) and second (**B**) molars at embryonic day 14.5 (E14.5; **A**) and E18.5 (**B**,**C**). Parasagittal (**D**,**E**) and frontal (**F**) sections across developing incisors from an E16.5 embryo (**D**), newborn (**E**) and a 3 day postpartum (dpp) pup (**F**). Inset in **E** is a magnified view of the boxed area in **E**. Parasagittal sections across molars at 12 dpp (**G**,**H**). **G′** and **H′** are magnified views of the boxed areas in **G** and **H**, respectively. The *ShhGFPCRE/R26R* and *K14-CRE/R26R* teeth share similar distribution patterns of CRE activity at 12 dpp. The only difference in the distribution of β-galactosidase activity between the two genotypes resides in the oral epithelium which exhibits the expected patchy pattern in the *ShhGFPCRE/R26R* jaws (**G**) and the expected homogeneous pattern in the *K14-CRE/R26R* jaws (**H**). dAM, differentiating ameloblasts; DP, dental papilla mesenchyme; EK, enamel knot; HF, hair follicles; IDE, inner dental epithelium; MA, maturation-stage ameloblasts; OE, oral epithelium; PL, papillary layer; SA, secretory ameloblasts; SI, stratum intermedium. Scale bars: 500 μm (**G**,**H**), 200 μm (**C**,**D**,**E**,**G′**,**H′**) and 100 μm (**A**,**B**,**F**).

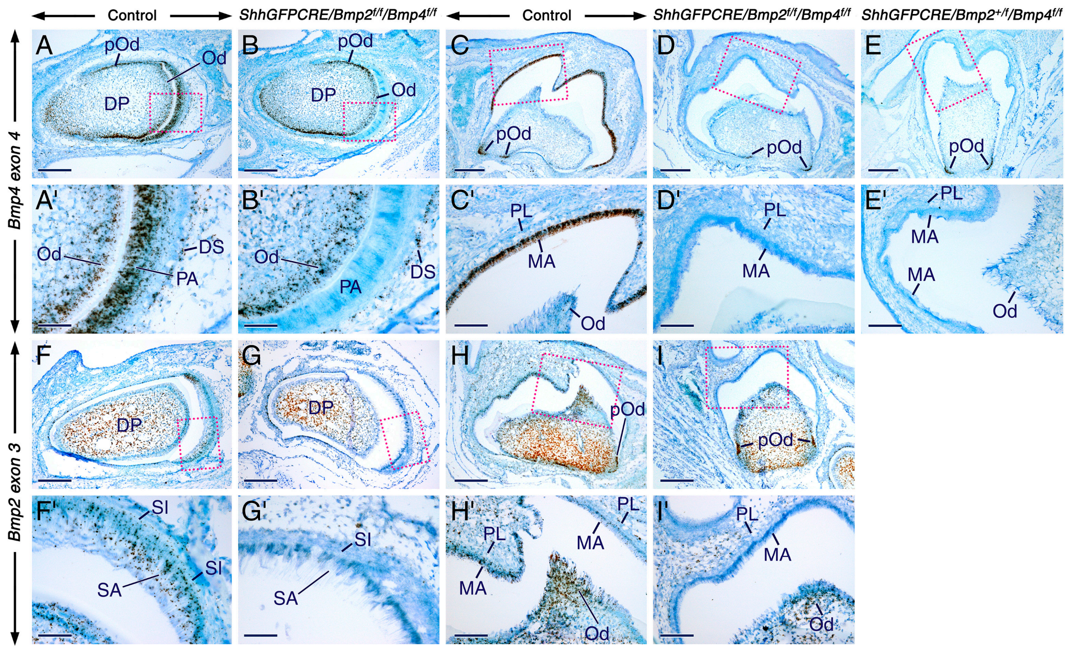

Figure S2. Deactivation of the *Bmp2* and *Bmp4* genes in the dental epithelium. **(A–I)** Representative RNAscope in situ hybridization with probes targeting the deleted sequences in the *Bmp4* (**A**–**E**) and *Bmp2* (**F–I)** genes in sections across incisors (**A**,**B**,**F**,**G**) and molars (**C**–**E**,**H**,**I**) from control (**A**,**C**,**F**,**H**), *ShhGFPCRE*/$Bmp2^{f/f}$/$Bmp4^{f/f}$ (**B**,**D**,**G**,**I**) and *ShhGFPCRE*/$Bmp2^{+/f}$/$Bmp4^{f/f}$ (**E**) mice at 12 days postpartum. The hybridization signals appear as dark brown dots. **A′**–**I′** are magnified views of the boxed areas in **A**–**I**. The mutant teeth exhibit irreversible loss of *Bmp2* and *Bmp4* expression in preameloblasts, stratum intermedium, secretory ameloblasts and maturation-stage ameloblasts and show the expected normal expression of these genes in cells derived from the dental mesenchyme such as preodontoblasts, odontoblasts, dental papilla/pulp cells and cells of the dental sac. DP, dental papilla/pulp mesenchyme; DS, dental sac mesenchyme; MA, maturation-stage ameloblasts; Od, odontoblasts; PA, preameloblasts; PL, papillary layer; pOd, preodontoblasts; SA, secretory ameloblasts; SI, stratum intermedium. Scale bars: 250 µm (**C**–**E**,**H**,**I**), 200 µm (**A**,**B**,**F**,**G**), 100 µm (**C′**–**E′**,**H′**,**I′**) and 50 µm (**A′**,**B′**,**F′**,**G′**).

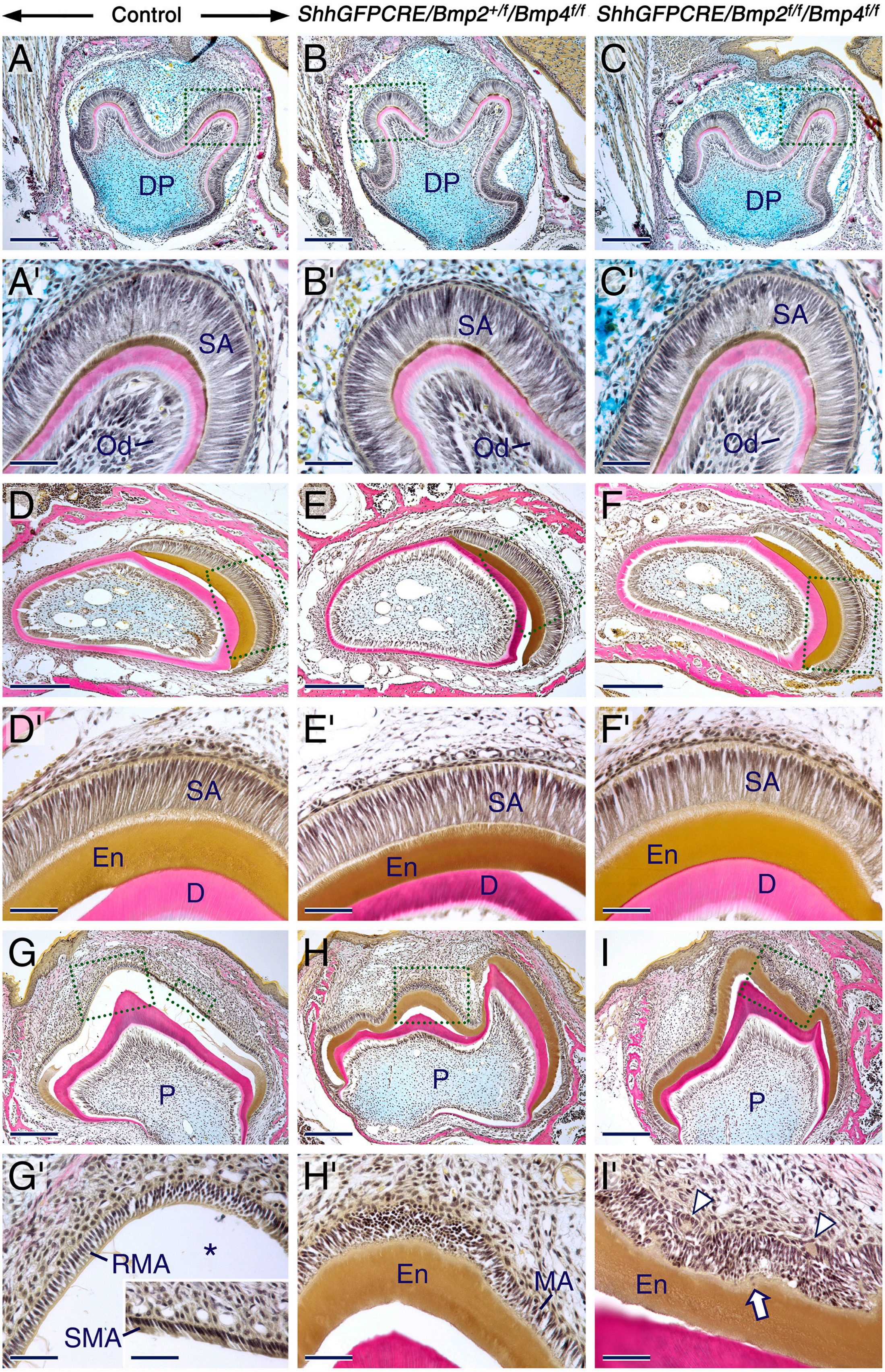

Figure S3. Combined loss of BMP2 and BMP4 signaling has no effects on tooth morphogenesis and enamel secretion but causes defects at the maturation stage of enamel formation. **(A–I)** Representative alcian blue van Gieson staining of sections across teeth from

control (**A**,**D**,**G**), *ShhGFPCRE/Bmp2*$^{+/f}$*/Bmp4*$^{f/f}$ (**B**,**E**,**H**) and *ShhGFPCRE/Bmp2*$^{f/f}$*/Bmp4*$^{f/f}$ (**C**,**F**,**I**) mice. **A′–I′** are magnified views of the boxed areas in **A–I**. Sections across 1 day post-partum (1 dpp) molars (**A–C**) showing normal tooth morphogenesis in the mutant teeth. Sections across 12 dpp incisors (**D–F**) at the level of the secretory stage of enamel formation showing normal development of secretory ameloblasts in the mutants. Sections across 12 dpp molars (**G–I**) showing abnormal maturation stage ameloblasts and persistence of the enamel matrix in the mutant teeth, indicating abnormal enamel maturation. The asterisk in **G′** indicates the enamel space left by the mature enamel after demineralization. Unlike the control teeth, which exhibit ruffle-ended (RMA) and smooth-ended (SMA) maturation-stage ameloblasts (**G′** and inset in **G′**), the mutant teeth show a dysplastic maturation-stage ameloblast layer with severely dysmorphic cells, some of which migrate away from the ameloblast layer and secrete an extracellular matrix (arrowheads in **I′**). The mutant teeth also exhibit cellular fragments, including nuclei, embedded in the enamel matrix (arrow in **I′**), and the retained enamel layer is wavy. Note the abnormal attachment of the apical border of mutant maturation-stage ameloblasts to the enamel matrix (**H′**,**I′**). D, dentin; DP, dental papilla mesenchyme; En, enamel matrix; P, dental pulp; SA, secretory ameloblasts; RMA, ruffle-ended maturation-stage ameloblasts; SMA, smooth-ended maturation-stage ameloblasts. Scale bars: 200 μm (**A–I**) and 50 μm (**A′–I′**).

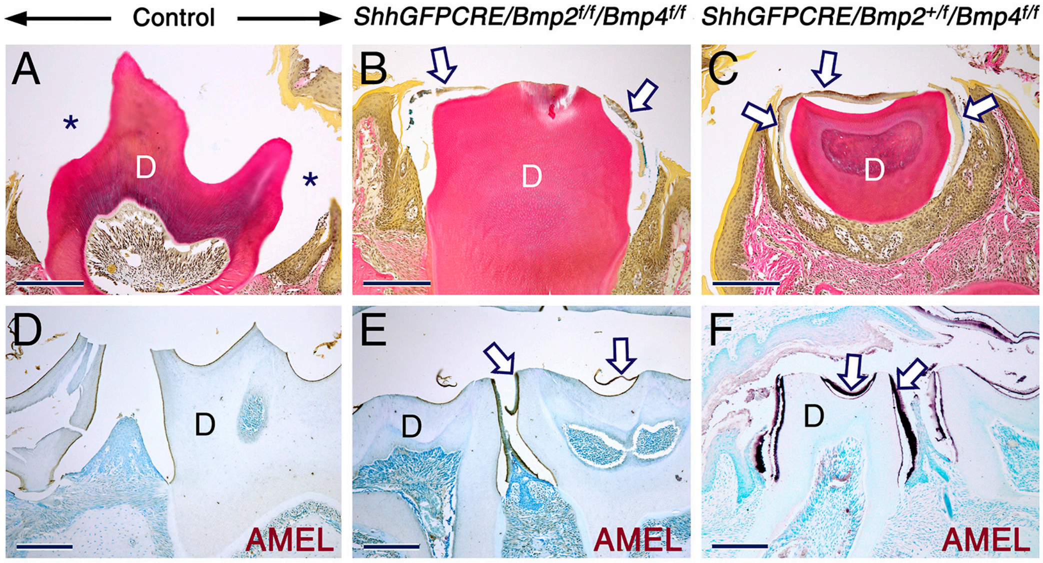

Figure S4. Severe dental wear and retained Amelogenin-containing enamel matrix in teeth with combined loss of epithelial BMP2 and BMP4 signaling. (**A–F**) Sections across erupted teeth from adult control (**A**,**D**), *ShhGFPCRE/Bmp2*$^{f/f}$*/Bmp4*$^{f/f}$ (**B**,**E**) and *ShhGFPCRE/Bmp2*$^{+/f}$*/Bmp4*$^{f/f}$ (**C**,**F**) mice. (**A–C**) Representative alcian blue van Gieson staining of frontal sections across molars. The mature enamel in the control tooth normally disappears after demineralization, leaving a space (asterisk in **A**). The mutant molars are severely abraded and display persistent enamel matrix (arrows in **B** and **C**), indicating abnormal enamel maturation. (**D–F**) Representative Amelogenin (AMEL) immunostaining (brown or purple) in parasagittal sections across molars showing retained Amelogenin-positive enamel matrix in the mutant teeth (arrows in **E** and **F**). D, dentin. Scale bars: 200 μm (**A–F**).

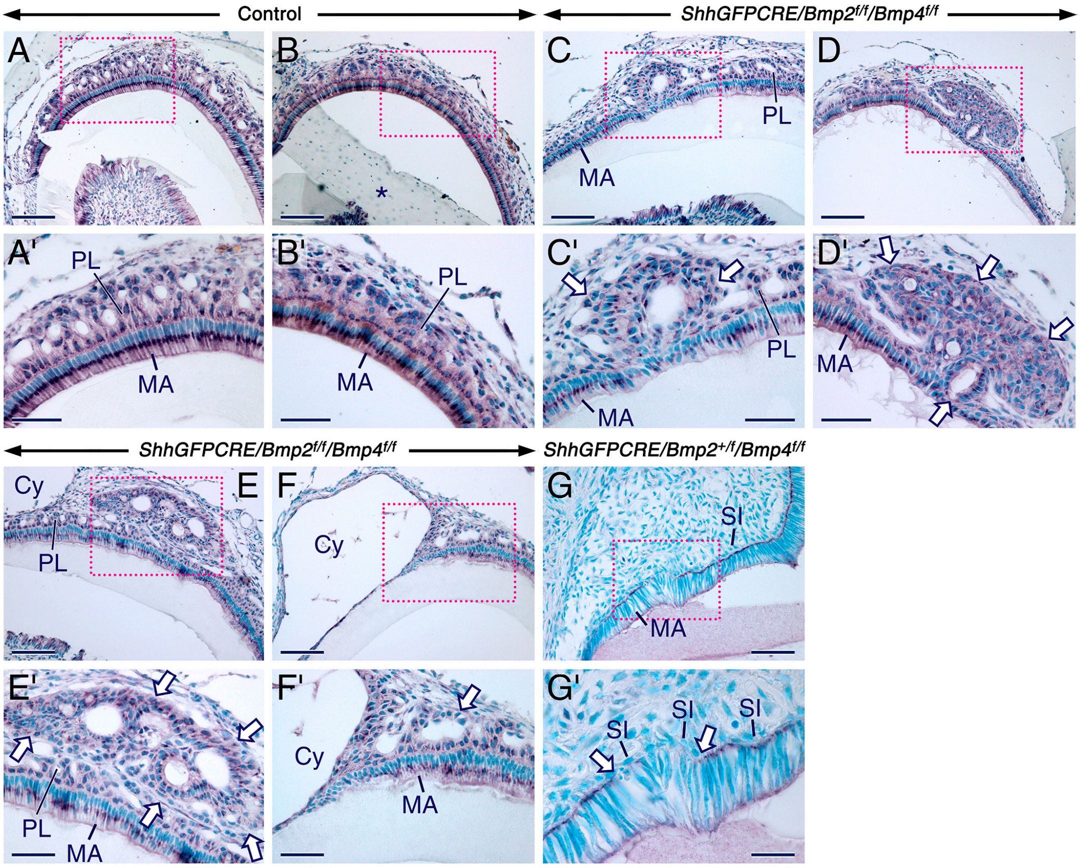

Figure S5. Maturation-stage ameloblasts with combined loss of BMP2 and BMP4 signaling emigrate away from the ameloblast layer and form cysts as well as tumor-like and gland-like structures. (**A–G**) Sections across teeth from control (**A**,**B**), *ShhGFPCRE/Bmp2$^{f/f}$/Bmp4$^{f/f}$* (**C–F**) and *ShhGFPCRE/Bmp2$^{+/f}$/Bmp4$^{f/f}$* (**G**) mice. **A′–G′** are magnified views of the boxed areas in **A–G**. Asterisk in **B** indicates artefactual presence of the jaw bone section over the tooth section as a result of its dislodgment during histological processing. Anti-Bax-immunostained sections across adult incisors (**A–F**) each taken at the level of the maturation-stage (**A**,**C**,**E**) and the late maturation-stage (**B**,**D**,**F**) of amelogenesis. The mutant incisors show a severely dysplastic maturation-stage ameloblast layer, and subsets of ameloblasts formed tumor-like and gland-like structures (arrows in **C′**,**D′** and **E′**) as well as cysts (Cy; **E**,**F**,**F′**). Note the abnormal attachment of the apical border of the mutant maturation-stage ameloblasts to the enamel matrix (**C′**,**D′**,**E**,**F′**). Anti-Bax staining (brown) was used as we found that it highlights the cells better than "normal" histology. Note that similar to previous findings in other cell types [Popgeorgiev, N.; Jabbour, L.; Gillet, G. Subcellular localization and dynamics of the Bcl-2 family of proteins. *Front. Cell Dev. Biol*. **2018,** *6*, 13], in dental cells, including maturation-stage ameloblasts, Bax immuno-staining concentrates in subcellular sites, likely in the endoplasmic reticulum and Golgi apparatus. (**G**,**G′**) Anti-ZO1-immunostained (dark purple) section across a molar from a 12 days post-partum mutant showing loss of ZO1 immunostaining between the basal pole of maturation-stage ameloblasts and the stratum intermedium. Note that at this site (demarcated with two arrows in **G′**) subsets of ameloblasts seem to have begun emigration from the ameloblast layer and the remaining ameloblasts are abnormally attached to the enamel matrix. Cy, cyst; MA, maturation-stage ameloblasts; PL, papillary layer; SI, stratum intermedium. Scale bars: 100 μm (**A–F**), 50 μm (**A′–F′**,**G**) and 20 μm (**G′**).
